# Neighbourhood Environment Correlates of Physical Activity: A Study of Eight Czech Regional Towns

**DOI:** 10.3390/ijerph8020341

**Published:** 2011-01-28

**Authors:** Dagmar Sigmundová, Walid El Ansari, Erik Sigmund

**Affiliations:** 1 Center for Kinanthropology Research, Faculty of Physical Culture, Palacky University in Olomouc, Tr. Miru 115, Olomouc 77111, Czech Republic; E-Mail: erik.sigmund@upol.cz; 2 Faculty of Applied Sciences, University of Gloucestershire, Oxstalls Campus, Oxstalls Lane, Gloucester GL2 9HW, UK; E-Mail: walidansari@glos.ac.uk

**Keywords:** Yamax pedometer, physical activity, ANEWS, number of steps, BMI, environment, neighbourhood, Czech population

## Abstract

An adequate amount of physical activity (PA) is a key factor that is associated with good health. This study assessed socio-environmental factors associated with meeting the health recommendations for PA (achieving 10,000 steps per day). In total, 1,653 respondents randomly selected from across eight regional towns (each >90,000 inhabitants) in the Czech Republic participated in the study. The ANEWS questionnaire assessed the environment in neighbourhoods, and participants’ weekly PA was objectively monitored (Yamax Digiwalker SW-700 pedometer). About 24% of participants were sufficiently active, 27% were highly active; 28% participants were overweight and 5% were obese. Although BMI was significantly inversely associated with the daily step counts achieved only in females, for both genders, BMI was generally not significantly associated with the criterion of achieving 10,000 steps per day during the week. Increased BMI in both genders was accompanied with a decline in participation in organized PA and with increasing age. As regards to the demographic/lifestyle factors, for females, more participation in organized PA was significantly positively correlated with the achieved daily step counts. In contrast, older age and higher BMI (for females) and smoking (for males) were significantly negatively correlated with the achieved daily step counts. In terms of the environmental aspects, pleasant environments were significantly positively correlated to daily step counts for both genders. Additionally, for males, better residencies (more family homes rather than apartment blocks) in the neighbourhood were significantly positively correlated with their daily step counts. For females, less accessibility of shops and non-sport facilities (depending on walking distance in minutes) were significantly negatively correlated to the achieved daily step counts. Individuals who lived in pleasant neighbourhoods, with better access to shops and who participated in organized PA (≥2 times a week) tended to meet the recommendations for health-enhancing PA levels. The creation of physical activity-friendly environments could be associated with enhancing people’s achieved daily step counts and meeting the health criteria for PA.

## 1. Introduction

Adequate amounts of physical activity (PA) have well documented positive effects on health [1–3]. However, despite concerted efforts aimed at increasing the PA levels of inhabitants of the European Union (EU), concerns have been raised that two thirds of people in the EU remain insufficiently physically active in terms of meeting recommended health guidelines [4]. In 2007, 40%–87% of the EU population reported a sedentary lifestyle [5], despite of the links between physical inactivity and overweight and obesity. For instance, in the Czech Republic, 55% of adults did not meet the health-enhancing guidelines for PA [6], with a corresponding 64.5% and 49% of adult men and women, respectively, being either overweight or obese [7]. By health-enhancing guidelines, we mean achieving 10,000 steps per day which is a recognized criterion for improving health. Indeed, adult women who walked 10,000 steps daily were likely to meet the health criteria of the American College of Sport Medicine [8]; and research also supported the view that achieving 10,000 steps a day represented PA that is accompanied by health benefits [9–11].

Over the past decade, interventions to increase daily PA levels have primarily focussed on motivational and educational initiatives designed to increase PA [12]. Yet, theoretical models of PA need to also account for a range of environmental factors (e.g., aesthetics, parks, paths, stores, *etc.*) and their interaction with other determinants (e.g., age, smoking, family, socio-economical status, *etc.*) [13]. Given that a body of PA research is premised on the ecological model [14,15] that argues that PA behaviour is dependent on psychological, demographic and environmental factors, there is a growing need to better understand the range of environmental factors that are associated with PA [15].

Many of the current interventions seek to create PA-enhancing settings that could increase PA across the population with long lasting effects [12]. For instance, environments that are appropriate for and conducive of walking were associated with higher PA and lower occurrence of overweight and obesity [16–18]. Similarly, the availability of sufficient numbers of pedestrian and cycling paths enhanced PA through active transportation [16,19,20]. Cities typically accommodate large segments of the population, with the attending services, shops, industries and entertainment avenues all packed on disproportionately small regions of land [21]. With the exception of sport areas, parks and historical centers, the majority of cities, with their densely populated areas, are usually barely suitable for daily PA [21]. Hence, identifying the environmental factors associated with PA behaviours of city inhabitants is an important strategy that supports meeting the health-enhancing criteria of PA.

The creation of PA-friendly environments is an approach that could boost human activity in developed countries [19]. Thus the ecological model is regarded by many as a potential solution that could overcome the challenges of overweight and obesity by increasing the PA achieved by people and decreasing their use of cars and other passive means of transportation [14]. Such a point of view highlights the importance of maintaining and supporting PA-friendly environments. Although the Czech Republic is a member of the International Physical Activity and the Environment Network) [22–25], to date, the relationships between PA and the quality of neighborhood environment in big Czech towns have not been documented. This is despite the calls for the necessity of research that addresses the health and PA-related environments of inner city neighborhoods [26]. Further, whilst questionnaires are usually employed for population-representative samples in order to estimate the amount of PA achieved by people, the use of pedometers for an objective estimation of daily PA is preferred [27]. Indeed, researchers have recommended the use of objective monitoring rather than questionnaires for the assessment of PA [27,28].

The Czech Republic and the other post-communist countries seem to be replicating the decreasing PA and increasing overweight and obesity patterns that have been previously observed in western countries. Hence effective public health policies could reduce the development of negative PA attitudes in the Czech Republic. The current study bridges this gap in the literature to assess a range of socio-environmental factors and examine whether such factors are associated with pedometer-determined PA. This is a critical aspect in planning healthy public policies.

### 1.1. Aims of the Study

This cross sectional study identified, across eight regional towns in the Czech Republic, the socio-environmental factors that are associated with meeting health guidelines for PA. The five specific objectives were to:

- describe the proportion of inhabitants across eight Czech regional towns by their pedometer-determined PA (number of daily steps) that is achieved on weekdays and weekend;
- describe the proportion of inhabitants of these towns by their BMI (computed from self-reported height and weight);
- assess the correlations between socio-environmental factors and achieved daily number of steps for males and females;
- examine the socio-environmental factors that are associated with achieving PA guidelines during the week (7 days) and during the working days; and,
- investigate the association between organized PA, overweight and obesity and their relationships to age in males and females.

## 2. Methods

### 2.1. Ethics

The current study was undertaken in the Czech Republic after approval by the Institutional Research Ethics Committee at Palacky University. Participation was voluntary and participants received no incentives and could withdraw from the study if they wished. Participants (adults) were provided with information about the aims, objectives and methods of survey before the start of monitoring of their PA. Data were anonymous and confidential and data protection was observed at all times. Each participant signed an informed consent for inclusion in the study.

### 2.2. Sample

Eight of the nine largest regional towns across the Czech Republic were selected (each between 90,000–380,000 inhabitants). Each selected town represented a delineated region within the Czech Republic: (Brno—Southern Moravia; Olomouc—Central Moravia; Ostrava—Northern Moravia; České Budějovice—Southern Bohemia; Hradec Králové and Liberec—Eastern Bohemia; Plzeň—Western Bohemia; and Ústí nad Labem—Northern Bohemia). Hence the whole of the Czech Republic was represented. The capital (Prague) was not included due to its specific features (e.g., much bigger size with >1 million citizens, in contrast to other regional towns that were selected) within the Czech Republic.

### 2.3. Instruments

We employed the Neighbourhood Environment Walkability Scale-Abbreviated (ANEWS) questionnaire [29] to assess the quality of environment in neighbourhoods [23,30]. The Czech version of questionnaire was developed in accordance with the Guide to Cultural Adaptation and Translation of the IPAQ Instruments [31]. First, the English version of the questionnaire was translated into Czech by three independent translators. Back translation of the final Czech version was then undertaken by three independent bilingual people. After the back translation, a bilingual expert group compared the Czech and back-translated English version in order to ensure that the translation was suitable for Czech culture and understandable for monolingual Czech participants.

The final Czech questionnaire was developed after this expert panel and comprised 53 items about the environment in neighborhoods, categorized into several sections: (A) types of residence in one’s neighbourhood (five items, e.g., “How common are townhouses or rows of houses of 1–3 storeys in your immediate neighbourhood?”); (B) stores, facilities and other things in one’s neighbourhood (23 items, e.g., “How long would it take to get from your home to the nearest businesses or facilities like supermarket, post office, library, bank, school *etc.*”); (C) access to services (six items, e.g., “Stores are within easy walking distance of my home”, “Parking is difficult in local shopping areas”, *etc.*); (D) streets in one’s neighbourhood (three items, e.g., “The streets in my neighbourhood do not have many cul-de-sacs”); (E) places for walking and cycling (four items, e.g., “There are sidewalks on most of the streets in my neighbourhood”); (F) neighbourhood surroundings (three items, e.g., “There are trees along the streets in my neighbourhood”); and, (G) neighbourhood safety [nine items, e.g., “The speed of traffic on most nearby streets is usually slow (30 mph or less)]. Responses to these questions were rated on ordinal scales 1–4 (1 = ‘strongly disagree’; 4 = ‘strongly agree’); or alternatively on 5-point Likert scales (1 = ‘none’; 5 = ‘all’). Participants also provided demographic and lifestyle information e.g., gender, age, height, weight, educational level, number of children, availability of driving licence, ownership of motor vehicle/s, smoking, participation in organized PA and some general information.

The objective measurement of PA was undertaken as a seven-day monitoring using Yamax Digiwalker SW-700 pedometer (Yamax Corporation, Tokyo, Japan). Participants wore the pedometers on either the right or left side of their waist all day except for hygiene (e.g., showering, bathing) and swimming (the device is not waterproof). As pedometers are most accurate in estimating the number of steps and least accurate in estimating energy expenditure [32], the number of steps achieved was employed to express participants’ achieved PA.

### 2.4. Procedures and Participants

The survey and monitoring of PA were undertaken during Spring and Autumn 2007 (seasons with comparable weather conditions in the Czech Republic). We used Microsoft Excel to randomly select addresses in each city. Trained research coordinators visited each selected household, and family members >15 years of age were informed about the study’s aims/objectives and asked if they wished to participate in the study. If in agreement, each participant signed an informed consent, completed the questionnaire and participated in the PA monitoring. The initial sample comprised 1,652 participants, out of which only 67% (n = 1,107) completed the ANEWS questionnaire appropriately and also completed the week-long monitoring using the Yamax SW-700 pedometer. Another 13.4% of the sample (n = 221) were also excluded due to incomplete or contradictory information in their questionnaires, or due to short daily periods of PA monitoring using the pedometers (<10 hours per day). In the subsequent randomization of participants according to age categories, another 237 individuals were excluded from the analysis as they were either: (1) <18 years of age (current analysis focused only on adults); (2) were randomly excluded because some of our age subgroups comprised slightly more proportions of people when compared to the proportions of the population with similar age as provided by the Czech Statistical Office concerning the population distribution using statistical software; and, (3) some respondents did not live in the cities selected for the study and thus were also excluded. Hence the current analysis included 649 participants (376 females; 273 males) aged 18–67 years.

### 2.5. Statistical Analysis

Statistical analysis was undertaken using Statistica 8.0 and SPSS 17.0 software. Spearman correlation coefficient quantified the associations between the environmental, demographic and lifestyle variables, and daily number of steps achieved. In order to compute any significant differences between the number of steps achieved on working days and on weekend; or between number of steps performed by men and women, ANOVA test and coefficient of effect size *d = (M_1_–M_2_)/SD_pooled_* were used, in accordance with others [33].

On the basis of logistic regression analysis (Forward Stepwise LR method), respondents were divided into two groups: those meeting or not meeting the criterion of achieving 10,000 steps per day. The model also included environmental factors: e.g., accessibility of shops and sport facilities assessed on the basis of walking distance (≤ to 10 minutes—better accessibility, >10 minutes—worse accessibility); safety (less safe with a score < 0.6, safer with a score ≥ 0.6), where the score was created using IPEN scoring protocol [34], and by assigning values ranging from 0–1, while the answers were assigned on the basis of weighted and arithmetic means. The same procedure was used to create a variable relating to the environment (neighbourhood surroundings) that classified it as ‘pleasant’ environment (score < 0.6) or ‘unpleasant’ environment (score ≥ 0.6). Other demographic/lifestyle variables included e.g., the family situation (living alone/living in a family); ownership of a car (yes/no); smoking (yes/no); education (basic, middle school, high school); number of children (0, 1, 2, 3, 4 and more); monthly income (low <20,000 CZK [770 EUR], middle 20,000 CZK [770 EUR]— 34,000 CZK [1,308 EUR], and high ≥35,000 CZK [1,346 EUR]); age (age brackets 18–24, 25–34, 35–44, 45–54, ≥55 years old); organized PA (not participating in PA, participating once a week, or ≥2 times a week); and Body Mass Index (BMI, kg/m^2^). Coefficient of Nagelkerke R^2^ was computed as part of the regression analysis to provide information about the models’ goodness of fit. In this paper, we present the results of the regression models of the whole sample on working days and for the whole week. We do not report the results of the regression analysis for weekend days as its Nagelkerke R^2^ was very close to zero.

## 3. Results

### 3.1. Characteristics of the Sample

The analysis comprised data from 649 participants: 273 (42%) males and 376 (58%) females. Participants’ age ranged from 18 to 69 years (mean 36.29 years, SD = 13.04). One-fourth of the participants reported university education, 68% had graduated from high school, and 7% achieved only primary education. Based on self-reported height and weight that was used to compute participants’ BMI, more than half the sample (54%) reported normal weight (20 ≤ BMI < 25 kg/m^2^), followed by those who were overweight (BMI ≥ 25, 28%) and 5% were obese (BMI ≥ 30 kg/m^2^). About one third of respondents were either overweight or obese. Roughly 13% of participants were underweight (BMI < 20 kg/m^2^). A third (34%) of the sample lived alone, 34% lived with their family (without children) and 33% of participants lived with their family and children. Two thirds (66%) of participants did not have any children, whilst 20% of adults had one child and another 12% had two children (only 2% of participants reported having > 2 children). Most (84%) participants had a driving license, and the same proportion (84%, not exactly the same individuals) owned a vehicle. Monthly income was not reported by about a quarter (26%) of respondents. Roughly 25% of adults reported a monthly income of < 20,000 CZK (770 EUR), followed by those (29%) with income between 20,000 CZK (770 EUR)–34,000 CZK (1,308 EUR). About 17% reported an income between 35,000 CZK (1,346 EUR)–59,000 CZK (2,269 EUR), and only 2% of adults had monthly income > 60,000 CZK (2,308 EUR).

### 3.2. PA Activity and BMI across 8 Czech Regional Towns

Employing the pedometer-recorded number of daily steps achieved, and using published guidelines [11], 4% of our sample exhibited a ‘sedentary’ lifestyle, 16% were ‘little active’, 29% ‘somewhat active’, 24% ‘sufficiently active’, and 27% ‘highly active’. On average, females accomplished 10,790 steps per day (SD = 3,944) on weekdays and 9,644 steps per day (SD = 4,579) on weekend; whilst males achieved 11,093 steps per day (SD = 4,076) on weekdays and 10,063 steps per day (SD = 4,705) on weekend.

### 3.3. Correlation between Socio-Environmental Factors and Daily Number of Steps Achieved

Correlation analysis by gender (Table 1) showed significant associations between daily step counts and a range of factors. As regards the environmental aspects, for both genders, pleasant environment was significantly positively correlated to daily step counts that were achieved. Additionally, for males, better types of residences in their neighbourhood (*i.e.*, high prevalence of family houses rather than blocks of flats with >six storeys) were also significantly positively correlated to daily step counts. For females, less accessibility of shops and non-sport facilities (based on the walking distance in minutes) were significantly negatively correlated to the achieved daily step counts.

In connection with demographic/lifestyle factors, for females, more participation in organized PA was significantly positively correlated with the achieved daily step counts. In contrast, older age and higher BMI (for females) and smoking (for males) were significantly negatively correlated with the achieved daily step counts. Generally, the correlations were of low magnitude.

### 3.4. Socio-Environmental Factors Associated with the Achievement of PA Guidelines

Due to the identified significant differences between the number of steps achieved on working days and at weekends (F = 48.983; *p* < 0.001; d = 0.24), analysis of the associations of PA (achieving 10,000 steps per day) with socio-environmental factors was computed separately for the total weekly (7 days) number of steps (Table 2), and also for working days (5 days, Table 3). For the whole week (7 days), the results of the logistic regression (Table 2) showed that people living in pleasant neighbourhoods, with better accessibility to shops and those who participated in organized PA (≥2 times a week) were more likely to meet the health recommendations for PA (meeting the health criterion of achieving 10,000 steps per day). Conversely family situation, safety of the environment, accessibility of sport facilities, car ownership, smoking status, education level, number of children, monthly income, age and BMI were not significantly associated with the criterion of achieving 10,000 steps per day during the week (hence data not presented in Table 2).

For working days (5 days), the results of the regression analysis (Table 3) showed that participants who lived in pleasant neighbourhoods were ≈1.4 times more likely to achieve 10,000 steps per day in comparison to those living in ‘unpleasant’ neighbourhoods. With longer distances to the shops and services, participants were about one third less likely to meet the criterion when compared to those who lived in neighbourhoods with better accessibility (closer distances) to services. Similarly, those who regarded their neighbourhoods as safe and did not own a car were, on average, about twice as likely to achieve 10,000 steps per day as those who reported living in unsafe neighbourhoods and those who owned a car. Respondents participating in organized PA at least twice a week were about twice (1.84) more likely to achieve 10,000 steps per day when compared to those not participating in any organized PA. Conversely family situation, safety, accessibility of sport facilities, smoking, education, number of children, monthly income, age and BMI were not significantly associated with the criterion of achieving 10,000 steps per day on working days (hence data not presented in Table 3).

### 3.5. Association between Organized PA, Overweight and Obesity and Their Relationship to Age in Males and Females

Statistically significant differences in the achieved number of steps in relation to age were identified only in women (F = 2.874; p = 0.02).

**Figure 1.**
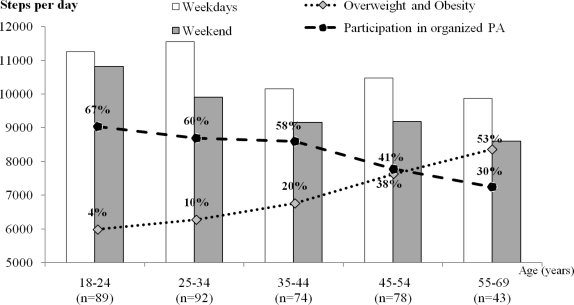
Association between age-related daily number of steps, participation in organized PA, and increases in overweight and obesity in females.

**Figure 2.**
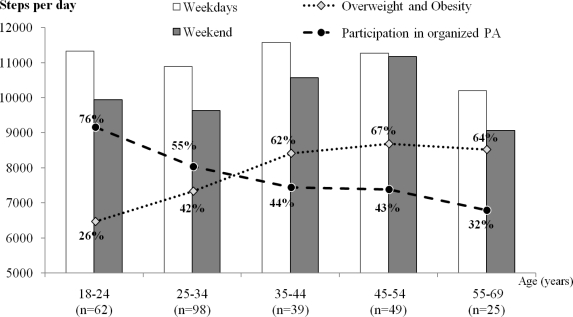
Association between age-related daily number of steps, participation in organized PA, and increases in overweight and obesity in males.

Figure 1 shows that for women, there was a slight step-wise decline in the participation in organized PA with the increase in age, where those in the oldest age bracket who participated in organized PA were about half of those in the youngest age bracket. This was simultaneously accompanied by a corresponding increase in overweight and obesity with increasing age. For men (Figure 2), the highest decrease in the participation in organized PA was after 25 years of age, and it markedly decreased until the age of 44. Between the age brackets 18–24 and 35–44 years, men’s participation in organized PA declined by 32%.

## 4. Discussion

PA-friendly environment is seen to support human activity [19]. However, the majority of PA and environment-related research that is based on country-representative samples has been mostly undertaken in the USA, Australia or Western Europe [13,35,36]. Even though the Czech Republic is a member of International Physical Activity and Environment Network, to date, the relationship between PA related environment and pedometer-determined PA in big cities in the Czech Republic has not been well documented. Hence the current study focused on PA-related environment in big Czech regional towns. We employed correlation and regression analyses to explore the social and environmental factors that were associated with meeting the health criterion for PA (achieving 10,000 steps per day).

As regards the first two objectives of the study, we described the levels of PA and BMI across a sample of 8 Czech towns. To the best of our knowledge, this is the first study to undertake this task. A proven inverse relationship between leisure time PA and BMI supports that PA and physical inactivity could be key determinants of the increasing overweight and obesity trends in Western countries [37]. Highly physically active people were twice less likely to become obese in comparison to those who were the least physically active [37]. Likewise, individuals who were sedentary for ≥35 hours a week in their leisure time, were 68% more likely to become obese than those individuals who were sedentary for ≤15 hours per week [37]. An apparent trend in the decrease in PA has been also identified in a wide population study in the U.S.A., where across 18 years, obesity had increased by 8%, and regular PA decreased from 53% to 43% [38]. In our sample (eight Czech towns), we found that about 33% were overweight and obese, 4% of people reported sedentary lifestyle, and 16% of people were ‘little active’ (inactive). An international World Health Study undertaken in 2002–2003 [39] argued that in the Czech Republic, 7–10% of people were inactive (or little active) as opposed to the European average where two-thirds of EU inhabitants were insufficiently physically active [4]. This suggested that the Czech Republic could still be doing well in terms of PA. Similarly, the International Prevalence Study on Physical Activity undertaken in 2002–2004 confirmed that the Czech Republic (along with New Zealand, USA, Canada and Australia) were among the nations with prevalent high levels of PA [40]. Given that walking comprises almost 40% of PA in the Czech Republic [40], such high ‘walkability’ of the Czech population should be maintained and needs to be supported with appropriate environments that are conducive to such healthy activities.

In connection with the third objective, we assessed the correlations between socio-environmental factors and the daily number of steps achieved by individuals. Our findings suggested that socio-environmental factors (pleasant environment, types of residences in neighbourhood, accessibility of shops and non-sport facilities) exhibited significant positive associations with the daily numbers of steps. However, these associations were not of very large magnitude. Environmental interventions could support PA [41], but environmental challenges such as the construction of more roads instead of pedestrian and cycling paths, increased mechanization at work, and insufficient number of green zones tend to significantly influence daily PA [12]. Our findings are in agreement with studies undertaken in Australia [42,43], Belgium [35] and Georgia—USA [36], where the aesthetics of the environment, good conditions for walking (paths, parks), and services and shops that are reachable within walking distance were all associated with walking. It could be that merely living in a walking-friendly environment increases the number of achieved steps per day or the amount of PA that is undertaken [16–18,25,44]. Indeed, research from Australia [19,43], Finland [45] and Japan [24] found that the creation of PA-friendly environments could increase PA across the population with long term effects [12].

Regarding the fourth objective, we examined the socio-environmental factors associated with achieving health-enhancing PA guidelines. Our study of a sample of young adults and adults aged 18–69 from across eight regional towns in the Czech Republic showed that a range of environmental variables were significantly associated with PA. These findings are in agreement with a study of adults using the IPAQ questionnaire (as in the current study) in Belgium that reported that the quality of sidewalks, good accessibility of shopping outlets and convenient facilities for physical activity outside the home were associated with physical activity [35]. Our findings are also in support of other research that found that aesthetics, safety, and opportunities for sports and physical activity were all related with physical activity [46–48]. Studies on PA and the environment are usually difficult to generalize and compare internationally: due to specific environmental conditions, culture and traditions, many features of the environment and PA behaviour are very unique across individual countries.

In order to better design programs that could increase PA to a level that enhances health, an understanding of the social and environmental factors that are implicated is necessary. In relation to socio-environmental factors, the current study showed that in city areas with pleasant and safe neighborhoods where shops and other services are better accessible, people were more likely to meet the health recommendation of 10,000 steps per day. Significant associations between the accessibility of shops and PA have been also confirmed by other researchers in Belgium [35], Japan [24] and Australia [19,42]. A Japanese study also reported that a ‘safe’ environment increased the chance of meeting the recommendations for PA [24]. Alternatively, as regards lifestyle factors, our findings suggested that people’s participation in organized PA at least twice a week and non ownership of a car increased the chances of achieving PA levels that met the health recommendations. Conversely, car ownership decreased the likelihood of achieving 10,000 steps per day. Our findings are in agreement with others: e.g., in Japan [24], using the ANEWS questionnaire (as in the current study), the factors that increased the chance of meeting recommendations for PA included good access to public transportation, availability of pedestrian and cycling paths, and not having a motor vehicle.

In connection with the fifth objective, we investigated the association between organized PA, overweight and obesity and their relationship to age in males and females. We found that with increasing age, both men and women reported a decreased participation in organized PA. This is in agreement with others [38,49] who reported an increase in overweight and obesity with increasing age. Age-related decline in PA is well documented [1,3,38]. However, the current study showed a noticeable decrease of PA with age only in women. The biggest decrease was after 35 years of age, probably due to having children and the raft of accompanying responsibilities of child care. Leisure time PA and sport participation (especially organized PA) have positive effects on the total PA and future PA, because for both genders, previous sports participation keeps individuals remaining more physically active [50,51]. Another point to note in the current study was that the participation in organized PA decreased with age more in men than it did in women. Concurrently we observed an earlier increase of overweight and obesity in men soon after the age of 35 years, whilst the substantial increases in overweight and obese women was around 55 years of age. Possible reasons for such findings would probably include the age-related decline of basal metabolism; mechanization and sedentary lifestyles [1,37]; the early termination of regular sport participation and organized PA in men; and conversely, more interest in body aesthetics in women [52].

This study has limitations. Individuals who agreed to be included in the study and who completed the week-long PA monitoring could likely be people from the more physically active proportion of the population. Therefore, the findings might reflect higher PA levels than there truly are. Although the study included eight regional towns, due to funding constraints, not all regional towns in the Czech Republic were included. Similar surveys need to be also undertaken in other Czech cities/towns. Most participants evaluated their environments of neighbourhoods as safe and walkable, and hence, the study could have missed the opportunity to undertake comparisons with the opposite extreme: environments that comprised unsafe neighborhoods with very low walkability. Further, using pedometers to assess weekly PA could reflect reactivity and motivation for participation in PA due to visual depiction of the number of steps [53]; future research could benefit from the use of motion sensors without display e.g., Actigraph [54]. The detailed analysis of PA-related environment in selected cities with application of Geographic Information Systems is also needed; as well as research that addresses PA related environment in different parts of cities such as in the centre, outskirts, and in high- and low-walkable environments. The use of geographic information system analyses would be essential in order to create designs for cycling and pedestrian paths and thus support the walking environment in the Czech Republic. Future research should also address the role of organized physical activity on PA habits throughout the life span; people’s preferences of organized PA and how offers of one’s preferred PA with good accessibility could be associated with actual higher PA levels; and the importance of instating active transport as an essential part of daily PA.

## 5. Conclusions

In our sample of eight Czech towns, more than half of the inhabitants exhibited adequate pedometer-determined levels of PA for maintaining one’s health. A third of the population was overweight or obese. For both genders, there was a decline in the participation in organized PA with increasing age which might need to be compensated for with accessible offers of physical activities for the elderly population. Women also exhibited significant differences in the achieved number of steps in relation to increasing age. Several social and environmental factors were associated with PA behaviour in these populations of regional Czech towns. Pleasant neighbourhoods, accessibility of shops and the participation in an organized PA at least twice a week were associated with increased chances of meeting the health-enhancing recommendation of 10,000 steps a day. However, on working days, the likelihood of achieving 10,000 steps a day was negatively associated with car ownership. The creation of physical activity-friendly environments can be associated with enhancing people’s achieved daily step counts and meeting the health criteria for PA.

## Figures and Tables

**Table 1.** Correlation between socio-environmental factors and daily step counts achieved across eight Czech regional towns by gender.

**Socio-environmental factors**	**r_S_–correlation**	
	**Female**	**Male**
	(n = 376)	(n = 273)
**Environmental factors**		

Pleasant environment in my neighbourhood	0.11 *	0.14 *
Safety of the environment	0.10	0.12
Walking-friendly environment	0.07	0.11
Types of residences in neighbourhood	0.03	0.22 **
Accessibility of shops and non-sport facilities (in minutes)	−0.12 *	0.04
Distance to sport facilities (in minutes)	−0.02	0.07
Accessibility of services in a neighbourhood	0.07	−0.06
Locations for walking and cycling	0.06	−0.05

**Demographic/lifestyle factors**		

Age	−0.17 **	−0.04
Highest achieved education	0.02	−0.12
Body Mass Index (kg·m^−2^)	−0.12 *	−0.05
Participation in organized PA (number of times per week)	0.18 **	0.11
Smoking	−0.03	−0.13 *
Family life (living in a family/alone)	0.09	−0.07
Number of children in the family	0.08	−0.05
Having driving license	−0.01	−0.09
Monthly income	−0.04	−0.09

* *p* < 0.05;

** *p* < 0.01.

**Table 2.** Achieving 10,000 steps per day during the week (7 days): Socio-environmental factors.

**Variable**	**n**	**%^a^**	**OR**	**CI**
Neighbourhood environment				

Unpleasant	353	46.5	Ref	
Pleasant	292	56.9	1.613**	1.167–2.230

Accessibility of shops (minutes of walking)				

Better accessibility	413	54.7	Ref	
Worse accessibility	230	46.5	0.703*	0.503–0.983

Participation in organized PA				

None	300	47.3	Ref	
Once a week	120	50.0	1.087	0.699–1.690
≥ 2 times a week	229	57.6	1.559*	1.087–2.235

% ^a:^ percentage of participants who met the criterion in a given area; OR: odds ratio; CI: 95% confidence interval; Ref: reference group;

* *p* < 0.05;

** *p* < 0.01; Nagelkerke R^2^ = 0.042.

**Table 3.** Achieving 10,000 steps per day on working days (5 days): Socio-environmental factors.

**Variable**	**n**	**%^a^**	**OR**	**CI**
Neighbourhood environment				

Unpleasant	353	53.3	Ref	
Pleasant	292	61.6	1.443*	1.036–2.010

Accessibility of shops (minutes of walking)				

Better accessibility	413	60.5	Ref	
Worse accessibility	230	52.2	0.711*	0.506–0.999

Neighbourhood Safety				

Less safe	49	40.8	Ref	
Safer	594	58.4	2.026*	1.096–3.745

Ownership of a car				

No	106	67.0	Ref	
Yes	543	55.4	0.572*	0.360–0.909

Participation in organized PA				

None	300	53.0	Ref	
Once a week	120	53.3	1.058	0.676–1.654
≥ 2 times a week	229	65.1	1.844**	1.266–2.686

% ^a:^ percentage of participants who met the criterion in a given area; OR: odds ratio; CI: 95% confidence interval; Ref: reference group;

* *p* < 0.05;

** *p* < 0.01; Nagelkerke R^2^ = 0.067.
